# Fluorescence enhancement of PbS colloidal quantum dots from silicon metasurfaces sustaining bound states in the continuum

**DOI:** 10.1515/nanoph-2023-0195

**Published:** 2023-06-15

**Authors:** Li Liu, Ruxue Wang, Yuwei Sun, Yi Jin, Aimin Wu

**Affiliations:** State Key Laboratory of Functional Materials for Informatics, Shanghai Institute of Microsystem and Information Technology, Chinese Academy of Sciences, Shanghai, 200050, China; Center of Materials Science and Optoelectronics Engineering, University of Chinese Academy of Sciences, Beijing, 100049, China; Centre for Optical and Electromagnetic Research and International Research Center for Advanced Photonics, College of Optical Science and Engineering, Zhejiang University, Hangzhou, 310058, China

**Keywords:** bound states in the continuum, fluorescence enhancement, metasurface, PbS quantum dot

## Abstract

PbS colloidal quantum dots (CQDs) can be considered a promising lighting material, but their emission performance is mired by defect sites, strong photo-induced activity, and interaction with the environment. Here, we utilize periodic silicon metasurface sustaining a symmetry-protected bound state in the continuum to enhance the near-infrared emission of PbS CQDs at room temperature. In the experimental investigation, it is observed that the fluorescence of the coated PbS CQDs is enhanced by 10 times by the fabricated metasurface, and the emission peak has a quality factor up to 251 at wavelength 1408 nm. Meanwhile, the potential of this work in sensing is demonstrated by showing that the enhanced emission is disturbed by the introduction of sparse gold nanoparticles. In all, this work confirms that dielectric metasurfaces sustaining bound states in the continuum can be adopted to efficiently improve the emission performance of PbS CQDs which may find various practical applications including on-chip silicon-based optical sources and integrated sensors.

## 1 Introduction

PbS colloidal quantum dots (PbS CQDs) have some fascinating advantages, the bandgap is wide and tunable, the reactive surface is large, and they can be fabricated by solution processability. They have been extensively applied in gas sensors [1–4], solar cells [5, 6], remote infrared imaging [7], and broadband photoelectric detectors [8], and also introduced into integrated photonic devices for on-chip light sources [9, 10]. However, PbS CQDs generally suffer from inefficient emission and poor radiation directivity. Semiconductor plasmonic nanocrystals have been used to enhance near-infrared fluorescence emission from PbS CQDs, making them act as more efficient and faster quantum emitters [11, 12]. However, the energy transfer to the free electron gas in the metal generates losses, which may lead to severe quenching of the fluorescence emission [13–15]. And, the large absorption loss of metals can severely limit the plasmonic applications in the near-infrared spectral range [16]. Compared to metal nanoresonators, dielectric ones are preferred since the material loss is negligible [17]. Some innovative methods have been raised to design resonant dielectric nanoresonators [18–23], but they are weak in confining optical energy, so that the corresponding quality factors (Qs) need to be improved.

Bound states in the continuum (BICs) supported by periodic dielectric photonic crystal slabs, also viewed as planar metasurfaces [24–27], may provide a preferable way for improving the emission performance of PbS CQDs. Guided modes are usually leaky so their *Q* factors are limited. However, under some special conditions, the leakage can be inhibited by symmetry protection or destructive interference between two resonant modes so that the guide modes become BICs of infinite *Q* values. BICs from symmetry protection, appearing at the center of the Brillouin zone (Г) [28, 29], are usually more stable than those from destructive interference so such BICs are discussed in this work. The potential of BICs is great in enhancing the interaction between light and matter, but they cannot be directly utilized since they are decoupled with the radiative continuum. Symmetry-breaking approaches, either by slightly breaking the excitation field symmetry or the structure symmetry, are required to make BICs applicable [30–32]. The excitable guided modes near BICs at point Г are leaky, but the leakage is rather weak so their *Q* values may be rather large, inverse to the degree of the deviation of their wave vectors from point Г. This point is utilized in many lasing applications [33–35]. And, in many nonlinear and sensing applications with external exciting sources, the structure symmetry is usually broken to make perfect BICs become quasi ones, whose *Q* values are inverse to the degree of the structure asymmetry [36–39].

Here, BICs are used to enhance the emission of PbS CQDs. A silicon (Si) metasurface supporting a symmetry-protected BIC is designed to enhance the emission of PbS CQDs. PbS CQDs will be coated on the fabricated metasurface to implement the fluorescence enhancement. Further, sparse gold (Au) nanoparticles are also randomly distributed on the QDs-coated metasurface to demonstrate the capability of the emission-enhancing system in sensing.

## 2 Results and discussions

**Figure 1:**
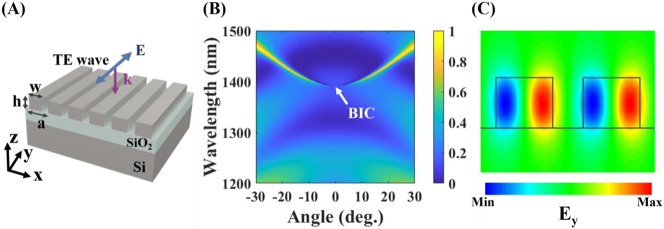
Metasurface consisting of a periodic array of Si bars. (A) Schematic of the metasurface. (B) Reflection as a function of the wavelength and the incident angle under TE polarization. A symmetry-protected BIC at Γ point is formed at *λ* = 1391 nm. (C) Electric field profile of *E*_*y*_ for the symmetry-protected BIC. Grey lines show the Si bar boundary.

A dielectric metasurface, consisting of a periodic array of subwavelength Si bars on a silica (SiO_2_) spacer with a semi-infinite Si substrate (*n*_Si_ = 3.48 and *n*_SiO2_ = 1.45), is depicted in Figure 1(A). The period is *a*, the width and height of the bars are *w* and *h*, respectively. Numerical investigation for the scattering response of the metasurface is carried out by the finite-difference time-domain method. The *x*–*z* plane is the incidence plane, and TE polarization is for the case that the incident electric field is along the axes of the Si bars while TM polarization is for the case that the incident magnetic field is along the axes of the Si bars.

Figure 1(B) shows the reflection spectra of the metasurface with period *a* = 540 nm and width *w* = 330 nm, resolved in both the wavelength and the incident angle, under TE polarization. The bright trails indicating strong reflection, correspond to the resonant excitation of the leaky Bloch guided modes. A symmetry-protected BIC is formed at the gamma point (Γ) in the Brillouin zone at *λ* ∼ 1391 nm. The appearing of this BIC is characterized by spectral narrowing and vanishing of reflection as the incidence angle approaches the normal [40]. The eigenmode of the BIC is simulated, and the electric distribution of *E*_*y*_ on the *x*–*z* cross-section of the metasurface is shown in Figure 1(C). One can see that the distribution of the electric field along the *x* direction is odd so the BIC cannot be coupled by external incidence. This BIC robustness in topology cannot be removed unless large changes are introduced to the geometric parameters of the metasurface [41]. The TM reflection is also simulated and shown in Supplementary Information, which does not show the existence of a BIC.

**Figure 2:**
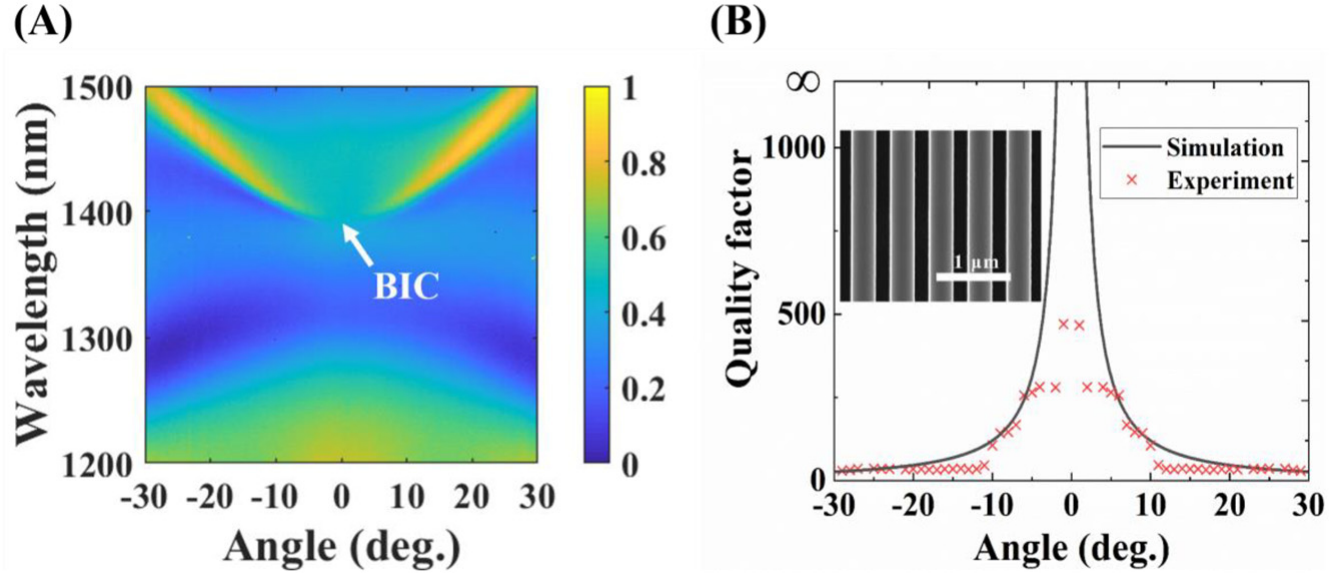
Experimental characterization of the fabricated metasurface. (A) Measured reflection as a function of the wavelength and the incident angle under TE polarization. (B) Angle-dependent *Q* values for the leaky guided modes neighboring the BIC at the same energy band. The dotted curve is from the experimental reflection, and the solid curve is from the calculated reflection. The inset is the SEM image of the fabricated sample.

The designed metasurface is fabricated based on a silicon-on-insulator (SOI) substrate (220 nm-thick top silicon layer, 3 μm-thick buried oxide layer, and Double-sided polished surfaces). The silicon metasurface is fabricated using electron beam lithography with positive photoresist (AR-P 6200). The photoresist pattern is then used as a mask to etch out the array of Si bars by inductively coupled plasma. The remaining photoresist is removed by 1-Methyl-2-pyrrolidinone (see Supplementary Information for more fabrication details). The fabricated metasurface has a footprint of 100 μm × 100 μm (about 185 periods), and the corresponding scanning electron microscope (SEM) image is shown in the inset of Figure 2(B). Then the reflection of the sample is measured by a microscopic angular-resolution spectral system (the noise signal is removed automatically). The TE-polarized reflection is shown in Figure 2(A). One can see that the measured result is well consistent with the simulated result (Figure 1(B)). And with the increase of the oblique incidence angle, the deviation between the measured and simulated reflections becomes obvious, which is because the adopted laser spot in the measurement has an area of about 100 μm^2^, which is comparable with the sample. The measured reflection can be used to calculate the *Q* value of the leaky guided mode in principle, but the fit is required to resolve the contribution of some neighboring resonant modes to the reflection (see Supplementary Information). Based on the Lorentzian fit method, the *Q* values of the guided modes neighboring the BIC on the same energy band are obtained and shown in Figure 2(B). As the incident angle approaches zero, the *Q* factor of the excited guide mode is dramatically increased. This tendency confirms the typical characterization of a BIC.

The fabricated metasurface is then applied to enhance the spontaneous emission of QDs. One can understand such emission enhancement based on the reciprocity theory [42]. It is first assumed that low loss is introduced into the silicon metasurface. The normal incidence does not excite the protected BIC so that the absorption from the metasurface is low since there is no strong localized electric field. If the loss is replaced with gain, the enhancement of the emission is weak. However, oblique incidence [43, 44] can excite the corresponding leaky guided mode near the BIC to produce a strong localized electric field so that the absorption from the metasurface is enlarged. Theoretically, the angle of oblique incidence is extremely close to 0° for the symmetrical structure, which is a precision that asymmetric structures cannot achieve. Then, if the loss is replaced with gain, there is an obvious enhancement in the emission. There exists an optimized leaky guided mode near the BIC that may induce the strongest emission enhancement. When QDs coated on the metasurface, the mechanism of the emission enhancement is similar. QDs usually carry out emissions in a finite bandwidth and a wide angular range. Although the symmetry-protected BIC cannot be directly used in the emission enhancement, the neighboring leaky guided modes of high Qs can be excited to strongly enhance the emission of QDs (numerically illustrated in Section 5 of the Supplementary Information).

**Figure 3:**
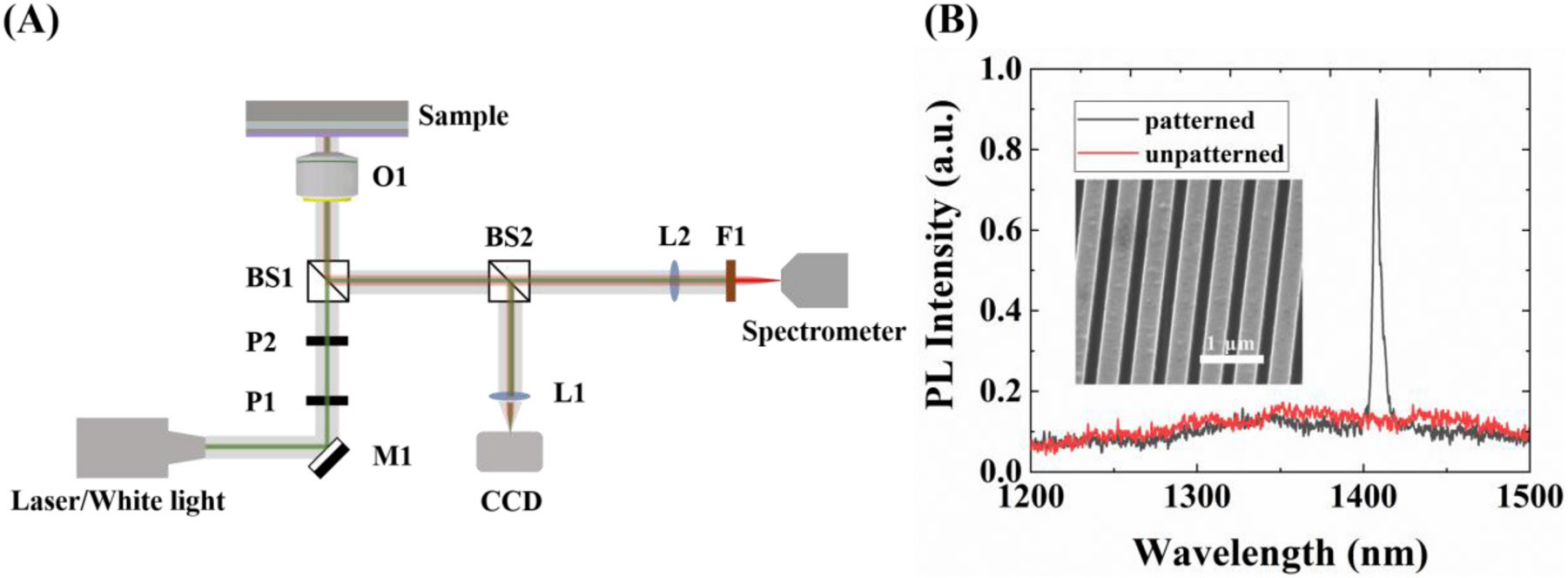
PL of the QDs-coated metasurface sample. (a) Optical setup for characterizing the micro-PL spectrum. M1, Silver mirrors; BS1, BS2, Beam splitters; P1, P2, Pinholes; L1, L2, Lens; O1, Objective lens; F1, Filter. (B) Measured PL spectra of the patterned metasurface (black curve) and unpatterned area on the same SOI chip (red curve). The inset is an SEM image of the QDs-coated metasurface sample.

The PbS CQDs are of a core–shell structure with PbS as the core and CdS as the shell layer. The surface is coated by hydrophobic ligand oleic acid and evenly dispersed in the *n*-octane solvent. The mixture solution is spin-coated on the top of the metasurface sample at 3000 rpm for 30 s and rinsed with ethanol at 2000 rpm for 20 s. The coated PbS CQDs form a thin film with a thickness of approximately 20 nm (*n* ∼ 1.63 and *k* ∼ 0 near 1400 nm) (see Supplementary Information). The SEM image of the QDs-coated metasurface is shown in the inset of Figure 3(B), where one can see that the PbS CQDs are evenly distributed on the surface of the sample.

The home-built micro-PL setup is shown in Figure 3(A). The white light is used to illuminate and position the grating, which can be switched to a pump laser. The pump laser beam at 532 nm is first expanded by a lens array and the first beam splitter to fill the aperture of the objective (5×, numerical aperture (NA) = 0.1) and focused onto the metasurface. The light beam from the metasurface is reflected through the first beam splitter into the following light path and then split by the second beam splitter into two beams, of which one is used for imaging (Sona, ANDOR) and the other is used to measure the PL. An 1100 nm long-pass filter is used to filter the pump laser and allow the collected fluorescence to reach the spectrometer (SR-500i, ANDOR). The measured PL is shown in Figure 3(B). A narrow PL peak appears at wavelength 1408 nm, of which the full width at half maximum is 5.6 nm and the corresponding *Q* is 251. For comparison, PbS CQDs are also coated on the unpatterned area outside the patterned metasurface on the SOI chip, and their measured PL is also shown in Figure 3(B) (red curve). It can be calculated that the PL of the PbS CQDs is enhanced by about 10 times with the assistance of the metasurface.

**Figure 4:**
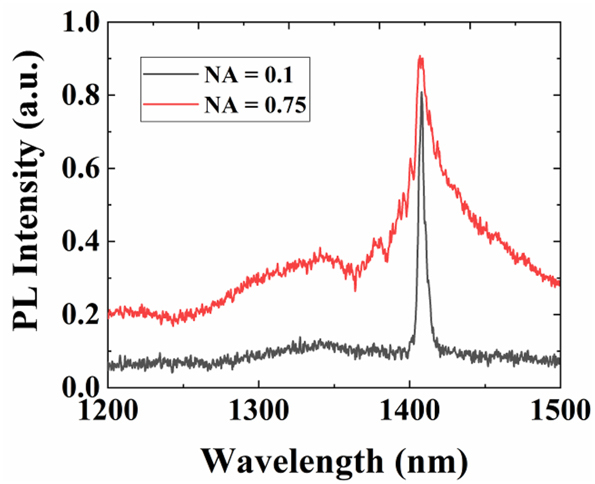
Measured PL spectra collected by different microscope objective lenses with NA = 0.1 (black curve) and NA = 0.75 (red curve), respectively.

Here, the influence of the adopted NA on the observed PL peak is investigated. If NA is small, the objective lens collect the PL within a small angular range (the aperture angle is about 11° for NA = 0.1) and the collected PL possesses a narrow bandwidth (see the black curve in Figure 4). If the NA is enlarged, the PL in a large angular range (the aperture angle is about 97° for NA = 0.75) is collected so that the measured PL peak is inhomogeneously broadened and the intensity is increased (see the red curve in Figure 4). The BIC is the fascinating focus point, and the neighboring leaky guided modes of small transverse Bloch vectors are the key point in the emission enhancement, thus the measured PL under NA = 0.1 is used to estimate the enhancement capability of the metasurface sustaining the symmetry-protected BIC [43].

**Figure 5:**
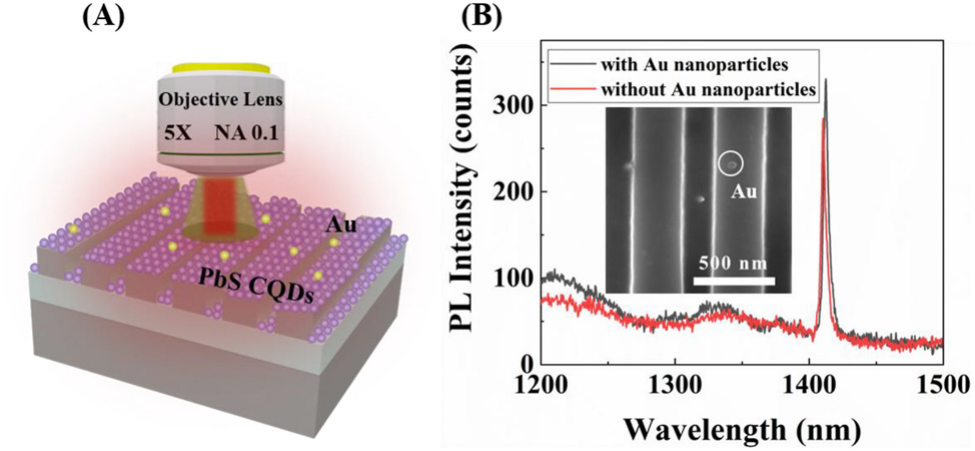
Sensing demonstration for the enhanced PL. (A) Schematic of the QDs-coated metasurface with sparse Au nanoparticles as a perturbation. (B) Measured PLs before (red curve) and after (black curve) introducing the Au nanoparticles. The inset is an SEM image of the fabricated sample with coated QDs and Au nanoparticles.

At last, a sensing prototype is demonstrated based on the enhanced PL. A small number of Au nanoparticles with a diameter of 60 nm are randomly distributed on the top of the QDs-coated metasurface as shown in Figure 5(A). The pump source is a continuous laser at 532 nm, and the emitted PL is collected by a 5× microscope objective lens. As can be seen in the inset of Figure 5(B), the Au nanoparticles are randomly dispersed on the metasurface with a surface density of about 4 per 1000 μm^2^. The PL of the sample with Au particles is then measured. It can be clearly observed in Figure 5(B) that the PL peak is redshifted from 1408 nm to 1410 nm when compared with that of the sample without Au particles. It should be noted that the Au nanoparticles couple weakly with the symmetry-protected BIC, but they can couple with the neighboring leaky guided modes. Thus, although the Au nanoparticles are sparse, they do disturb the emission property of the coated QDs. This indicates that the enhanced PL has potential in sensing since it is sensitive to the existence of sparse Au nanoparticles which can be replaced with other small objects, such as polystyrene microspheres.

## 3 Conclusions

In conclusion, we have proposed periodic silicon metasurface sustaining a symmetry-protected BIC, and efficiently enhanced the PL emission of the coated PbS CQDs. A *Q* factor of 251 at *λ* = 1408 nm is experimentally achieved, which is enhanced about 10 times than that of the CQDs sample without the artificial metasurface. The enhancement can be further improved by better fabrication accuracy, and the working wavelength can be tuned by a change of the geometry dimensions. One may also adopt merged BICs to reduce the degradation effect of the fabrication imperfection [45, 46]. The PL enhancement effect is also used for sensing, as is demonstrated by the clear redshift of the PL peak when introducing the Au nanoparticles. This passive structure of metasurface can be fabricated in a commercial CMOS platform with simple processes, thus it could be combined into silicon photonics integration for on-chip silicon-based light sources and nano-antennas, as well as fluorescent sensors.

## Supplementary Material

Supplementary Material Details
