# Supplementary material for: Fluorescence enhancement of PbS colloidal quantum dots from silicon metasurfaces sustaining bound states in the continuum
Source: Nanophotonics. 2023 Jun 15;12(15):3159–64. doi: 10.1515/nanoph-2023-0195 (PMC11501729; doi:10.1515/nanoph-2023-0195)
Supplement: Supplementary file 1 — Supplementary Material Details [file j_nanoph-2023-0195_suppl_001.pdf]

Supporting Information for

Li Liu[1,2], Ruxue Wang[1,2], Yuwei Sun[3], Yi Jin[3,4], Aimin Wu[1,2,5]

Fluorescence Enhancement of PbS Colloidal Quantum Dots from Silicon Metasurfaces Sustaining Bound States in the Continuum

1. State Key Laboratory of Functional Materials for Informatics, Shanghai Institute of Microsystem and Information Technology, Chinese Academy of Sciences, Shanghai, 200050, China

2. Center of Materials Science and Optoelectronics Engineering, University of Chinese Academy of Sciences, Beijing, 100049, China

3. Centre for Optical and Electromagnetic Research and International Research Center for Advanced Photonics, College of Optical Science and Engineering, Zhejiang University, Hangzhou, 310058, China

4.jinyi_2008@zju.edu.cn

5.wuaimin@mail.sim.ac.cn

1. Fabrication of the PbS CQDs-coated metasurface

The silicon metasurface coated with PbS CQDs is fabricated using standard nanofabrication techniques. First, we spin-coat 400 nm positive photoresist (AR-P 6200) on the top of an SOI wafer (220 nm-thick top silicon layer, 3 μm-thick buried oxide layer, and double-side polished). Then, we use electron-beam lithography to define the metasurface pattern on the photoresist. After that, the pattern is transferred to the subsequent inductively coupled plasma dry etch. After the remaining photoresist is removed, PbS CQDs (Xingzi, OA-11913-25, 25 mg/mL) are mixed with n-octane solvent. The mixture is spin-coated on the metasurface sample at 3000 rpm for 30 s and rinsed with ethanol at 2000 rpm for 20 s. At last, heating for 10 minutes at 80°C is performed to remove the excess organic solvent.

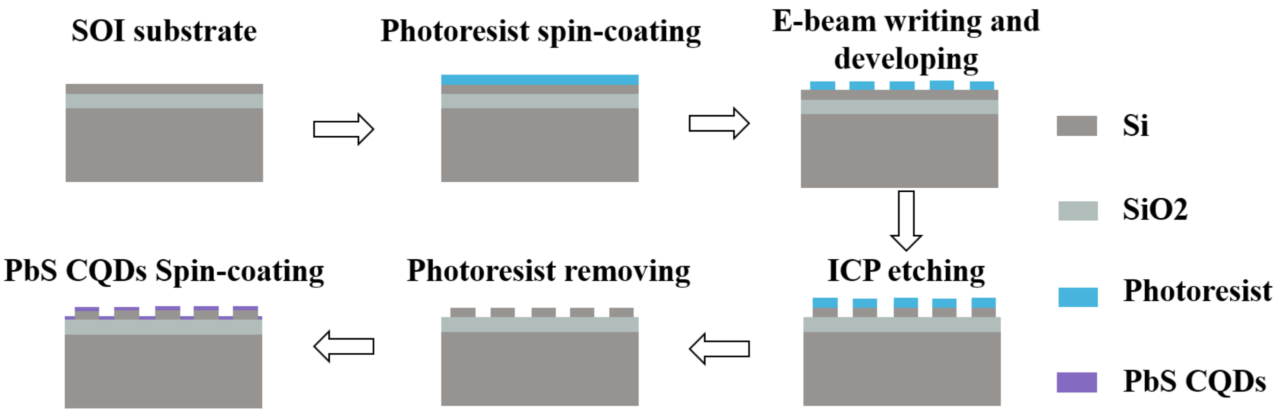

Fig. S1: Fabrication process of the silicon metasurface sample coated with PbS CQDs.

The fabricated metasurface with smooth surfaces and steep sidewalls is satisfactory, whose SEM images are shown in Fig. S2. However, the fabrication error can not be ignored and the sample size is

finite, which may degrade the concerned BIC to some degree. To make the practical BIC close to the ideal one, reducing the fabrication imperfection as much as possible and further enlarging the sample size are necessary.

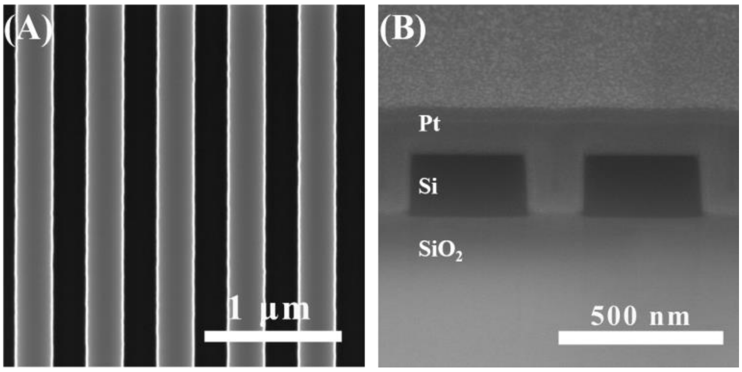

Fig. S2: SEM images of the fabricated silicon metasurface. (A) Overhead view. (B) Cross-section view.

2. Simulated and measured TM reflections of the designed metasurface

The simulated TM reflection of the designed metasurface is shown in Fig. S2(A), and the measured one of the fabricated metasurface is shown in Fig. S2(B). The TM reflection does not show the existence of a BIC.

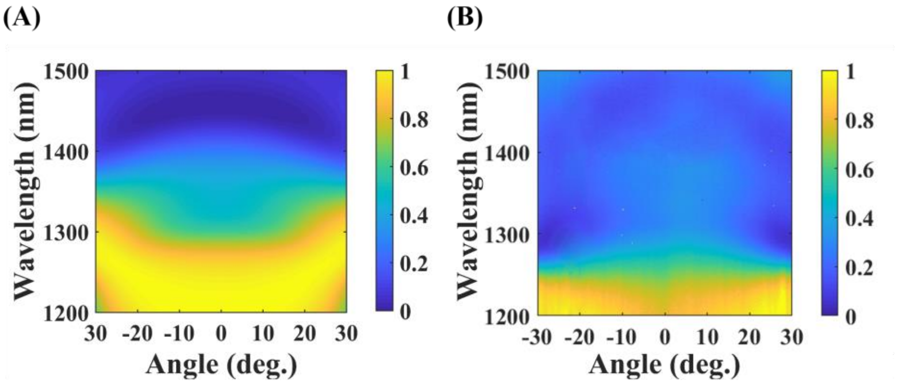

Fig. S3: TM reflection. (A) Simulated reflection of the designed metasurface, and (B) measured reflection of the fabricated metasurface.

3. Determining the Q factor of a leaky guided mode based on fitting the reflection

To illustrate the calculation process, the reflection spectrum at 6° is extracted from Fig. 2(A) as an example and shown in Fig. S4. Based on the Lorentzian fit method [1], this reflection curve is fitted with three Lorentz peaks (the two narrow peaks are from the excitation of two leaky guided modes and the one from the Fabry-Perot resonance of the metasurface as a slab). The Q factor for each Lorentz peak is easily defined now. Q = 251 is possessed by the narrowest Lorentz peak from the leaky guided mode near the BIC at the same energy band. It should be noted that this Q value is affected by the NA of the collecting objective lens (it is 0.1 here).

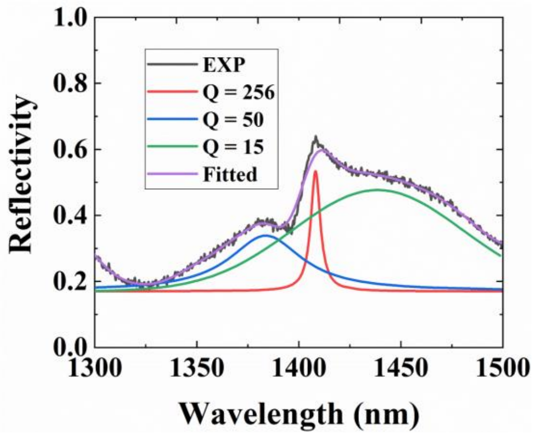

Fig. S4: Spectra fit for the reflection curve at 6° extracted from Fig. 2(A). The experimental reflection curve is fit by combining three Lorentz-shape peaks.

4. Spin-coated PbS CQDs

The SEM cross-section image of ten layers of PbS CQDs spin-coated on an SOI chip is shown in Fig. S5. The thickness is about 200 nm so that one can estimate that a single layer of PbS CQDs is approximately 20 nm.

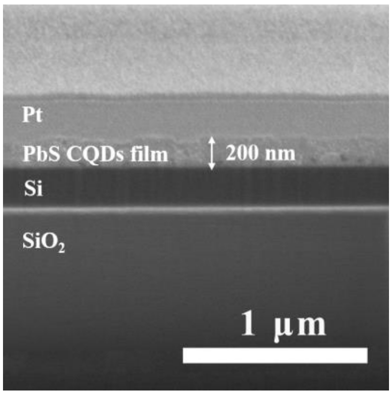

Fig. S5: SEM cross-section image of ten layers of PbS CQDs.

In the near-infrared band, the refractive index of a film of PbS CQDs is measured as shown in Fig. S6. The real part of the refractive index is about n = 1.63 and the imaginary part is k = 0 in the wavelength range of interest, so the PbS CQDs film can be regarded as a nondestructive material.

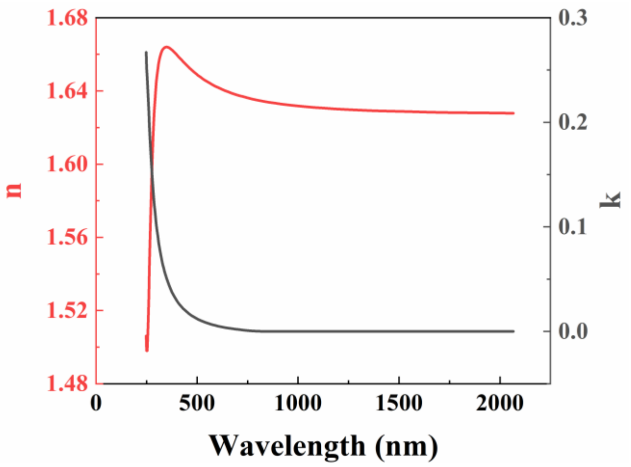

Fig. S6: Refractive index of a film of PbS CQDs.

The influence of the coated PbS QDs on the metasurface is also experimentally investigated as shown in Fig. S7. The reflection curve of the metasurface displays a redshift because of the introduction of the film of PbS CQDs [2].

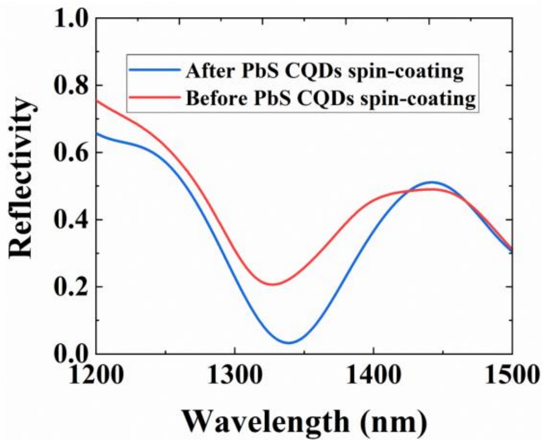

Fig. S7: Measured reflections before (red) and after (blue) spin-coating PbS CQDs on the metasurface sample.

5. Simulated emission enhancement of electric dipoles by the designed metasurface

Here, a numerical simulation is made for the emission enhancement of electric dipoles from the metasurface investigated in Fig. 1. A dipole is located at the center of the top surface of a Si bar in a unit cell (see Fig. S8(A)). In the simulation, the periodic boundary condition is adopted along the x direction. The orientation angle of the dipole is varied from 0° to 90° (the polarization of the dipole is parallel to the metasurface, and the orientation angle is defined as the angle between the dipole and the axes of the Si bars). The emitted power as a function of the wavelength and the orientation angle is shown in Fig. S8(B). At various orientation angles, the emission is always most enhanced at the same wavelength by exciting some preferable leaky guided mode close to the perfect BIC. The metasurface is anisotropic and sensitive to the polarization, thus the emission enhancement is variable for different orientation angles and it is weakest around the orientation angle of 90°.

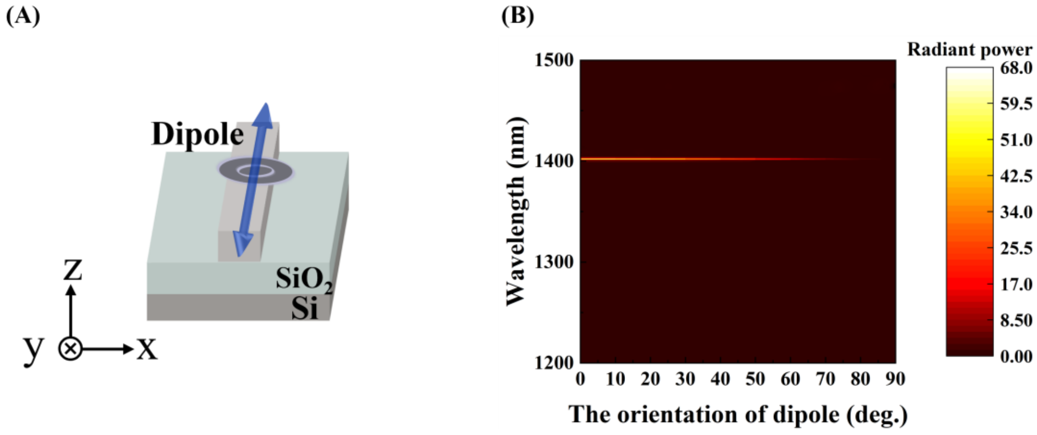

Fig. S8: Simulated emission enhancement of electric dipoles by the designed metasurface. (A) One dipole placed in a unit cell. (B) Emitted far-filed power as a function of the wavelength and the orientation angle.

Reference

[1] T. Mukoyama, “Fitting of Lorentzian to Mössbauer spectra by non-iterative method,” *Nuclear Instruments and Methods*, vol. 126, pp. 153-154,1975. https://doi.org/10.1016/0029-554X(75)90244-X

[2] Yang D, Zhou Q., “Solution-Processed P3HT:PbS-Based NIR Photodetector With FET Configuration,” *IEEE Photonics Technology Letters*, vol. 32, pp.77-80, 2020. https://doi.org/10.1109/LPT.2019.2957384
